# Unifocal Granuloma of Femur due to Langerhans' Cell Histiocytosis: A Case Report and Review of the Literature

**DOI:** 10.1155/2010/686031

**Published:** 2010-08-09

**Authors:** Harpreet Singh, Satnam Kaur, P. Yuvarajan, Nishant Jain, Lalit Maini

**Affiliations:** ^1^Department of Orthopaedics, Maulana Azad Medical College and associated Lok Nayak Hospital, Delhi 110002, India; ^2^Department of Pediatrics, Lady Hardinge Medical College and associated Kalawati Saran Children's Hospital, New Delhi 110001, India

## Abstract

The radiological diagnosis of osteolytic lesions of the long bones in pediatric population constitutes a challenge when the case history and clinical data are uncharacteristic. We believe that the description of few clinically and histologically proven cases to verify the existence of radiological signs useful for diagnosis may be of interest. Here, we describe a case of Langerhans' cell histiocytosis (LCH) presenting as unifocal eosinophilic granuloma of femur along with a brief review of the literature.

## 1. Introduction

Langerhans' cell histiocytosis (LCH) is a rare pediatric disease. The disease manifestations are varied and protean. A high index of suspicion is required for making an early diagnosis and initiating appropriate treatment, especially important for multisystem disease. Here, we report a case of LCH presenting with unifocal eosinophilic granuloma of femur and typical skin manifestations with probable central nervous system involvement.

## 2. Case Report

**Figure 1:**
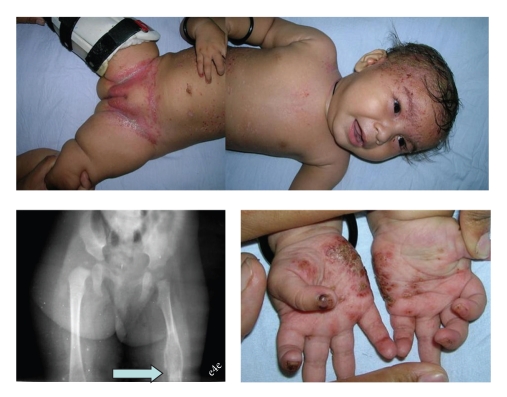
Picture showing the clinico-radiological presentation of the case.

An eight-year-old girl presented to us with complaints of diffuse swelling of the right thigh and skin rash. Her parents denied history of fever, swelling anywhere else, any systemic complaints, bleeding from any site, or trauma. On local examination, her right thigh was diffusely swollen and tender to touch. There were no signs of inflammation. No abnormal mobility was found in the underlying femur. General physical examination revealed scaly, erythematous, brown to red papular lesions over the scalp, abdomen, inguinal region, and palms (Figure 1). There was no pallor, icterus, lymphadenopathy, clubbing, edema, or swelling anywhere else. Rest of the general as well as systemic examination was unremarkable. X-ray of the limb showed a well-defined lytic expansile lesion in the diaphysis of femur with surrounding periosteal reaction (Figure 1). Rest of her investigations including complete hemogram, liver function tests, coagulation profile, skeletal survey, and chest radiograph were unyielding. Patient was treated conservatively by us with a functional brace while being investigated for the cause. The skin lesion biopsy revealed inflammatory lesion with numerous uni-, bi-, and multinucleated histiocytes in the background of numerous eosinophils and lymphocytes. The patient was subjected to bone biopsy which showed similar findings consistent with Langerhans' cell histiocytosis. The follow-up radiograph of femur at two weeks showed signs of healing of the lesion. However, two weeks after presentation, the child developed alteration of sensorium and started having recurrent seizures associated with symptoms of raised intracranial pressure. There was no associated fever, signs of meningeal irritation, or focal neurological deficit. She succumbed to her illness within 72 hours. MRI head could not be done due to very sick general condition of child and CSF study was not done because of raised intracranial pressure and suspected space occupying lesion. Postmortem lumbar puncture as well as autopsy was refused by parents which would have thrown light on underlying CNS pathology.

## 3. Discussion

Childhood histiocytosis constitutes an diverse group of disorders characterized by an intense proliferation of cells of monocyte-macrophage system of bone marrow origin. The writing group of the Histiocyte Society divided histiocytosis syndromes in children into three classes: class I is Langerhans' cell histiocytosis; class II (non-LC histiocytosis) includes the familial and virus-associated haemophagocytic syndromes, sinus histiocytosis with massive lymphadenopathy (Rosai-Dorfman), juvenile xanthogranuloma, and reticulohistiocytoma; class III consists of the malignant histiocytic diseases [1]. More recently, a revised classification schema included division into (1) dendritic cell disorders: Langerhans cell histiocytosis (LCH), secondary dendritic cell processes, juvenile xanthogranuloma, and solitary histiocytoma with a dendritic phenotype; (2) macrophage-related disorders: primary and secondary hemophagocytic syndrome, Rosai-Dorfman disease, and solitary histiocytoma with a macrophage phenotype; (3) malignant histiocytic disorders: monocyte related leukemia, extramedullary monocytic tumor, and dendritic cell or macrophage-related histiocytic sarcoma [2]. 

Langerhans' cell histiocytosis (LCH) occurs with an estimated incidence of about two to five cases per million yearly [3]. The three clinical variants of LCH include eosinophilic granuloma (EG), Hand-Schuller-Christian disease, and Letterer-Siwe disease. The condition is characterized by abnormal proliferation and dissemination of histiocytes, which are identical to the normal dendritic cells, first noted by Langerhans. The cause and pathogenesis of Langerhans' cell histiocytosis remain unclear, but the disease is believed to result from a disorder of immune regulation [4–6].

LCH has an extremely variable presentation. The skeleton is involved in 80% of patients and may be the only affected site especially in children >5 year of age [7]. EG has been reported to affect all the bones except those of the hand and feet [8–10]. The skull, femur, ribs, vertebrae, pelvis, long bones, and mandible are involved more commonly. Polyostotic-monostotic ratio cited in the literature averages 1 : 3 (range 1 : 2 to 1 : 6) [11–15]. Painful swelling is the most common initial sign. Proptosis from the lesion of orbital wall may be present. When mastoid process is involved, the findings can mimic mastoiditis. Extension to the middle ear can cause destruction of ossicles and deafness. EG of jaw bone is often associated with contiguous soft tissue swelling, “floating teeth,” gingival swelling, fracture, or pain. In spine, the lytic lesion can result in compression and collapse of vertebral body, causing vertebra plana. 

Cutaneous lesions, which may be present in association with bony involvement, frequently manifest as scaly, erythematous, seborrhea-like brown to red papules, especially pronounced in post auricular, axillary, inguinal, perineal areas, and over the scalp. The lesions may spread to involve the back, palms, and soles. About 50% of patients exhibit skin involvement at some time during the course of disease [7]. Other manifestations include localized/disseminated lymphadenopathy (33%), hepatosplenomegaly (20%), varying degree of hepatic malfunction, pulmonary and CNS involvement. 

Conventional analogical radiograms proved to be very suitable investigation, although use of digital technique has improved perception of details. In long bone, the lesion appears as an irregular lytic area in the medulla, usually with endosteal erosion. Marked cortical destruction may be evident and there may be an onion skin appearance due to periosteal reaction in diaphyseal lesions. Marginal sclerosis indicates that the lesion is healing. Long bone lesions are considered to be healed when the trabecular pattern has returned to normal or when lytic area had been totally replaced by a localized area of sclerosis. Flat bone lesions appear as irregular lytic areas. Either complete resolution may occur upon healing or lytic area may remain, though with a well-defined sclerotic margin [13, 15, 16]. In addition to radiography, other useful imaging techniques include skeletal scintigraphy (to identify any additional lesion, if any), Color Doppler (to rule out aneurysm), and magnetic resonance (to confirm a cystic lesion as fluid content is indicated by marked signal hypo intensity in T1 spin echo sequence and hyperintensity in T2 spin echo sequence) [17–19]. CT scan enables correct localization and can be used to plan the biopsy and treatment.

Diagnosis of LCH is confirmed by histological examination. The usual histopathological appearance is collections of eosinophilic polymorphs, always accompanied by histiocytes, and with varying proportion of lymphocytes, granulocytes, monocytes and plasma cells. Multinuclear giant cells of osteoclast type are occasionally present, but never in large numbers. Fibroblastic differentiation may be observed and is considered as evidence of healing. The diagnosis of Langerhans' cell histiocytosis is regarded as presumptive when the typical morphological characteristics of Langerhans' cells are seen with light microscopy and as designated when additional stains (e.g., protein S-100) are positive. Diagnosis is confirmed if stains for CD1a antigen are positive or when intracytoplasmic organelles (Birbeck granules) are seen with electron microscopy [4, 20, 21]. 

Organ dysfunction, rather than the extent of the lesions, is the most important prognostic feature and the aim of treatment is to correct such dysfunction as well as to limit the spread of the disease [4, 5]. The clinical course of single-system disease (usually bone, lymph node or, skin) is usually benign with a high chance of spontaneous remission. Therefore, treatment should be minimal. In contrast, multisystem disease should be treated with multiagent chemotherapy. Early diagnosis improves the response rate. Experimental therapies (indicated only for unresponsive disease) include immunosuppressive therapy with cyclosporine/antithymocyte globulin and certain new agents and modalities, such as imatinib, 2-chlorodeoxyadenosine, and stem cell transplantation. 

Currently accepted treatment modalities for EG include surgical curettage and filling the cavity with cancellous bone and intralesional steroids (for lesions in accessible areas) [9, 19, 21]. Also it responds well to low dose radiotherapy which is usually reserved for inaccessible areas. Most studies suggest that the dose should not be greater than 10 Gy [9, 22]. 

To conclude, LCH is a rare disease presenting in pediatric population. Nevertheless, it should be considered in differential diagnosis in children presenting with unifocal/multifocal osteolytic lesion especially in the presence of characteristic skin lesions. Consideration of important clinical clues may avoid delay in diagnosis which is an important predictor of response to treatment especially in multisystem disease. Characteristic imaging features described in this study may raise suspicion of LCH but are not sufficient for diagnosis. Appropriate histopathological studies are required to establish a diagnosis of LCH. While no standard of care exists for the treatment of LCH, management should be tailored to the individual patient based upon extent of disease, anatomic location, and radiographic extent of involvement.
